# A Machine Learning Model Based on PET/CT Radiomics and Clinical Characteristics Predicts Tumor Immune Profiles in Non-Small Cell Lung Cancer: A Retrospective Multicohort Study

**DOI:** 10.3389/fimmu.2022.859323

**Published:** 2022-04-29

**Authors:** Haipeng Tong, Jinju Sun, Jingqin Fang, Mi Zhang, Huan Liu, Renxiang Xia, Weicheng Zhou, Kaijun Liu, Xiao Chen

**Affiliations:** ^1^ Department of Nuclear Medicine, Daping Hospital, Army Medical University, Chongqing, China; ^2^ Department of Radiology, Daping Hospital, Army Medical University, Chongqing, China; ^3^ Chongqing Clinical Research Center for Imaging and Nuclear Medicine, Chongqing, China; ^4^ Advanced Application Team, GE Healthcare, Shanghai, China; ^5^ Department of Gastroenterology, Daping Hospital, Army Medical University, Chongqing, China

**Keywords:** positron emission tomography/computed tomography, radiomics, tumor immune microenvironment, lung cancer, machine learning

## Abstract

**Background:**

The tumor immune microenvironment (TIME) phenotypes have been reported to mainly impact the efficacy of immunotherapy. Given the increasing use of immunotherapy in cancers, knowing an individual’s TIME phenotypes could be helpful in screening patients who are more likely to respond to immunotherapy. Our study intended to establish, validate, and apply a machine learning model to predict TIME profiles in non-small cell lung cancer (NSCLC) by using ^18^F-FDG PET/CT radiomics and clinical characteristics.

**Methods:**

The RNA-seq data of 1145 NSCLC patients from The Cancer Genome Atlas (TCGA) cohort were analyzed. Then, 221 NSCLC patients from Daping Hospital (DPH) cohort received^18^F-FDG PET/CT scans before treatment and CD8 expression of the tumor samples were tested. The Artificial Intelligence Kit software was used to extract radiomic features of PET/CT images and develop a radiomics signature. The models were established by radiomics, clinical features, and radiomics-clinical combination, respectively, the performance of which was calculated by receiver operating curves (ROCs) and compared by DeLong test. Moreover, based on radiomics score (Rad-score) and clinical features, a nomogram was established. Finally, we applied the combined model to evaluate TIME phenotypes of NSCLC patients in The Cancer Imaging Archive (TCIA) cohort (n = 39).

**Results:**

TCGA data showed CD8 expression could represent the TIME profiles in NSCLC. In DPH cohort, PET/CT radiomics model outperformed CT model (AUC: 0.907 vs. 0.861, *P* = 0.0314) to predict CD8 expression. Further, PET/CT radiomics-clinical combined model (AUC = 0.932) outperformed PET/CT radiomics model (AUC = 0.907, *P* = 0.0326) or clinical model (AUC = 0.868, *P* = 0.0036) to predict CD8 expression. In the TCIA cohort, the predicted CD8-high group had significantly higher immune scores and more activated immune pathways than the predicted CD8-low group (*P* = 0.0421).

**Conclusion:**

Our study indicates that ^18^F-FDG PET/CT radiomics-clinical combined model could be a clinically practical method to non-invasively detect the tumor immune status in NSCLCs.

## Introduction

Immunotherapy has dramatically altered the traditional therapy strategies for cancers including melanoma, lymphoma, and non-small cell lung cancer (NSCLC) (1–3). Despite their breakthrough progress, only a subset of NSCLC patients has received clinical benefits (4, 5). Accumulating studies have revealed that tumor immune microenvironment (TIME) phenotypes mainly impact the efficacy of immunotherapy, especially CD8+ T cells infiltration into the tumors which is positively correlated with immunotherapy efficacy and survival (6–9). Knowing more about the TIME phenotypes in pre-treated NSCLC could help in identifying the patients who are more likely to respond to immunotherapy.

Currently, the gold standard for TIME phenotypes is based on the pathological result of biopsies or operations. However, there are several limitations to these methods to determine the immune status. First, these techniques are invasive, have risks, and are not suitable for some patients with severe conditions. Second, due to the tumor heterogeneity, the sampling error is relatively high by using these routine methods which rely on a single tumor sample (10–12). Third, tumor immune status would evolve dynamically during cancer therapy, which makes it difficult to determine the current TIME status from an archival sample (13, 14). Thus, it is necessary to develop non-invasive complementary approaches to reflect the entire and dynamic information of TIME.

Recent emergence of radiomics which contain a huge number of quantitative medical imaging features provides promising opportunities (15, 16). Compared with conventional imaging methods, radiomics can provide a more detailed characterization of tumor heterogeneity beyond the human eye, which reflects either macroscopic or cellular and molecular properties of tumor tissues (17–20). ^18^F-fluoro-deoxy-glucose positron emission tomography/computed tomography (^18^F-FDG PET/CT) is widely used in clinics to diagnose, stage, and monitor therapeutic efficacy in lung cancer. Recent studies have revealed that ^18^F-FDG PET/CT could show the metabolic status of the TIME (21–24). The metabolic pattern of ^18^F-FDG uptake in tumors could be highly associated with tumor-infiltrating immune cells (25). However, to our knowledge, there is no predictive model developed by using PET/CT radiomic features and clinical characteristics to predict TIME profiles in NSCLC.

In this study, we aimed to establish, validate, and apply a machine learning model to evaluate TIME profiles using ^18^F-FDG PET/CT radiomics data combined with clinical characteristics in cohorts of NSCLC patients from Daping Hospital (DPH) and The Cancer Imaging Archive (TCIA) dataset. We also evaluated whether CD8 expression signature could represent the TIME profiles by using the RNA sequencing (RNA-seq) data of lung squamous-cell carcinoma (LUSC) and lung adenocarcinoma (LUAD) from The Cancer Genome Atlas (TCGA) dataset.

## Materials and Methods

### Study Design and Data Collection

**Figure 1:**
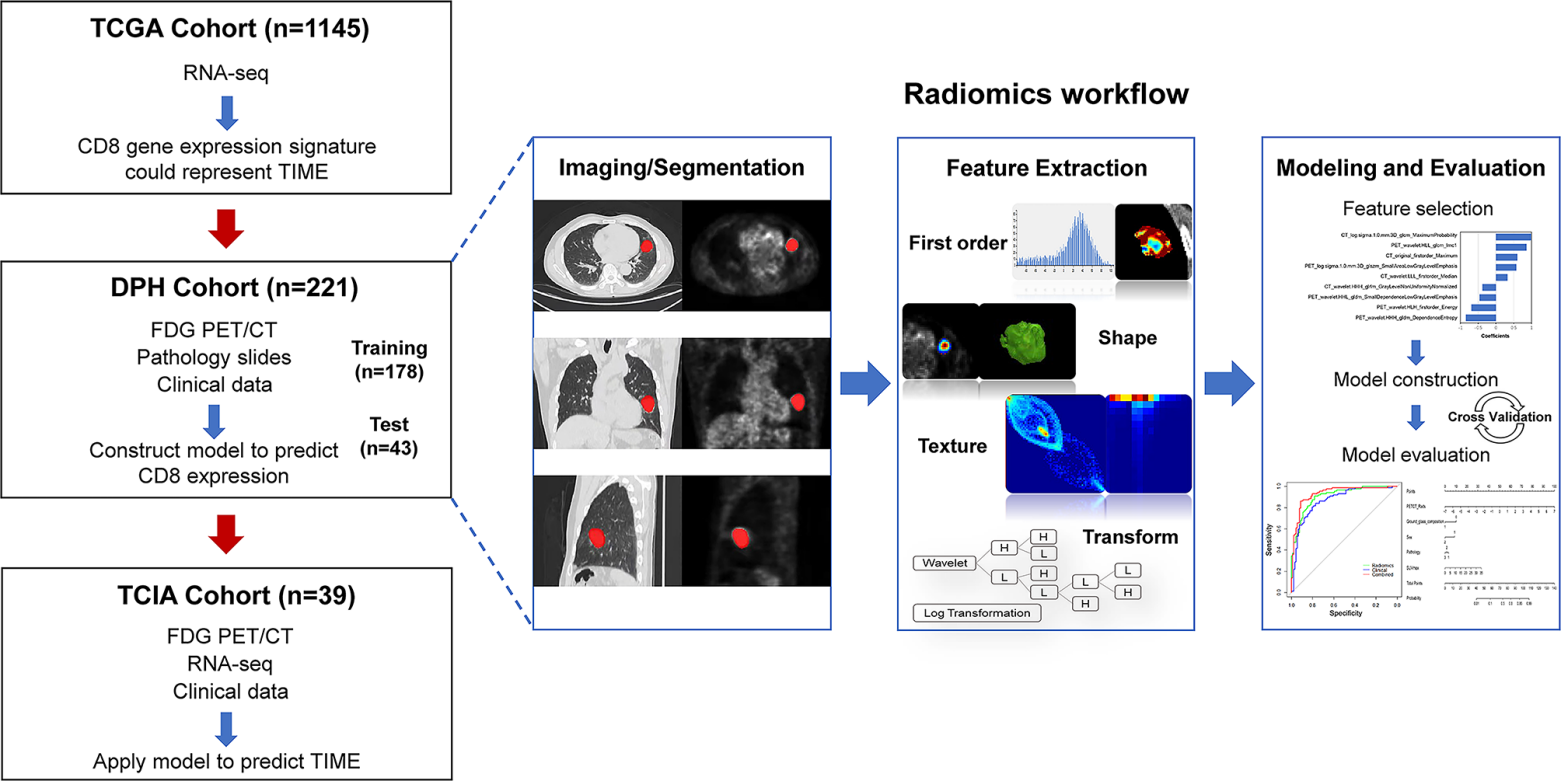
The workflow of overall study. Firstly, we evaluated whether CD8 expression signature could represent the TIME profiles by using the RNA-seq data in TCGA cohort (n = 1145). Then, machine learning model was trained and validated using DPH cohort (n = 221) with ^18^F-FDG PET/CT radiomics-clinical features to predict CD8 expression status. The model was then applied to predict TIME phenotypes in TCIA cohort (n = 39). The right row is the radiomics workflow.

In this retrospective multicohort study, radiomics analysis was performed in NSCLC patients from two independent cohorts, DPH dataset and TCIA dataset. TCGA dataset was used to evaluate whether CD8 expression could represent the TIME profiles in NSCLC. **Figure 1** depicts the overall study design.

The DPH cohort consisted of 221 patients who were pathologically confirmed NSCLCs. These patients underwent pre-therapy ^18^F-FDG PET/CT scan in our department from January 2019 to December 2020. Patients were classified into CD8-high and CD8-low groups, according to the pathological results (**Supplementary Material**). Inclusion criteria were as follows: (1) no anti-cancer therapy received before ^18^F-FDG PET/CT scan, (2) surgery or biopsy was performed within 2 weeks after ^18^F-FDG PET/CT scan, (3) patients were confirmed NSCLCs pathologically, (4) no history of other malignancy, and (5) a maximum diameter of the lesion > 1 cm to avoid partial volume effects. The exclusion criteria were as follows: (1) pure ground-glass nodule (pGGN) without FDG metabolism and (2) poor imaging quality. This retrospective study was conducted with the approval of the Ethical Committee of Daping Hospital, Army Medical University (No.2020055). This study’s use of human subjects complies with the Declaration of Helsinki.

### Choosing Target Gene Expression Signature

The RNA-seq data of 1145 NSCLC samples, information (gdc_manifest_20210810_002850) regarding the clinical data of these NSCLC patients were collected from the TCGA dataset (https://tcga-data.nci.nih.gov/tcga/). Perl software (http://www.perl.org/) was employed to organize the data. Although CD8A is known to represent CD8+ cytolytic T cells (CTLs) in the tumor microenvironment (TME), we first analyzed whether CD8A could represent the TIME profiles. The cut‐off values were set as log2 (fold change) > 2 for screening differentially expressed genes (DEGs) and adjusted *P* < 0.05 using the “DESeq2” and “limma” R packages (version 4.1.1). Samples from TCGA dataset were divided into two groups as follows: CD8-high group, CD8A expression higher than the median; CD8-low group, CD8A expression lower than the median. The protein–protein interaction (PPI) network from Metascape was obtained and reconstructed by Cytoscape 3.7.0 software. Functional enrichment was performed, including Gene Ontology (GO) terms that contained molecular function categories, cellular components, biological processes, and Kyoto Encyclopedia of Genes and Genomes (KEGG) pathway analysis. The highly associated modules and key genes in DEGs and differentially expressed immune genes (DEIGs) were identified by RNA‐seq analysis and weighted gene coexpression network analysis. Potential molecular mechanisms in these two groups were investigated by analyzing gene set enrichment analysis (GSEA) to acquire down- and up-regulated pathways. FDR <0.05 was defined as statistically significant.

### PET/CT Scan

All 221 patients in the DPH cohort were examined by Biograph 64 HD PET/CT (Siemens). Patients fasted for more than 6 hrs before scanning and had a blood glucose level < 8 mmol/L. Then, ^18^F-FDG was injected intravenously (3.7 MBq/kg, pH 5–7, radioactive purity > 95%). After 45-60 min, patients underwent PET/CT examination from the vertex to the proximal legs. CT scan parameters were 130 mA tube current, 120 kV voltage, and 5 mm slice thickness. Subsequently, whole-body PET scan was performed (4-6 bed positions at 90 s/bed position, FOV = 700 mm × 700 mm, matrix = 168 × 168, slice thickness = 5 mm) with correction for dead time, scatter, and decay. PET images were reconstructed by TrueX and fused with CT images.

### Segmentation, Feature Extraction, and Selection

The lesion was delineated by ITK-SNAP 3.8.0 software (www.itksnap.org). Two experienced radiologists, blinded to the clinical data of the patients, performed volume of interest (VOI) segmentation. For CT segmentation, VOI of lung cancers were drawn on lung window. For PET segmentation, a threshold of 40% SUVmax was used to delineate the VOI semi-automatically. Subsequently, the images were preprocessed by the Artificial Intelligence Kit (version 3.2.0, GE Healthcare). Images were preprocessed by resampling the isotropic voxel into 1 × 1 × 1 mm with linear interpolation. There were 874 radiomics features totally extracted from each VOI (VOI_CT, VOI_PET), including first order, shape, gray level co-occurrence matrix (GLCM), gray level size-zone matrix (GLSZM), gray level run-length matrix (GLRLM), neighborhood gray tone difference matrix (NGTDM), and neighboring gray level dependence matrix (GLDM). Advanced filters were applied with Laplacian of Gaussian (LoG, sigma 1.0 mm) and wavelet decompositions with all possible combinations of high (H) or low (L) pass filter in each of the three dimensions (HHH, HHL, HLH, LHH, LLL, LLH, LHL, and HLL). The 221 patients were stratified into training and validation groups randomly at 7:3. Feature standardization was performed before feature selection. Radiomics features were selected by using two algorithms. First, we removed the redundant and less-relevant features using the minimum-redundancy and maximum-relevance (mRMR). Then, the optimized feature subsets were selected by the least absolute shrinkage and selection operator (LASSO) method. The efficiency of model fitting and complexity was measured by Akaike Information Criterion (AIC).

### Clinical Feature Collection and Selection

The quantitative clinical features were age, gender, smoking history, pathology, stages, SUVmax, maximum length of tumor, leaflet, bur, air bronchogram, vacuole sign, ground glass composition, calcification, and pleural adhesion. All PET/CT imaging features were independently analyzed by two experienced radiologists who were blinded to the clinical data. The nominal variable was analyzed by chi square test or Fisher’s exact test. The continuous variable with abnormal distribution was analyzed by Mann-Whitney test, while, continuous variable with normal distribution was analyzed by t-test. Then, univariate logistic analysis was employed to investigate the discriminative clinical features between CD8-high (immune-inflamed, ‘hot’ TIME) and CD8-low (immune-desert, ‘cold’ TIME) groups.

### Model Construction

Three different radiomics models (PET/CT, PET, and CT radiomics models) were developed to evaluate TIME profiles in NSCLC. Then, Rad-score and clinical features were combined to establish a multivariate logistic regression model (combined model) and to develop a predictive nomogram for establishing a risk-scoring model. The nomogram’s calibration was assessed using calibration curves, which was confirmed by Hosmer–Lemeshow test. The diagnostic efficiency of the models in predicting TIME profiles of NSCLC was evaluated by receiver operating characteristic (ROC) curve.

### Machine Learning-Based TIME Prediction in TCIA Cohort

The combined model was applied to TCIA cohort, in which 39 NSCLC patients were performed ^18^F-FDG PET/CT scanning pretherapy. We selected the target lesions and performed segmentation and feature extraction as described before. We applied the radiomics-clinical combined model to divide into two groups. The difference in the immune scores was compared by t-test. DEGs in these two groups were identified by the ‘limma Bioconductor’ software package in R (version 4.1.0, available at http://www.R-project.org). The ‘ggplot2’, ‘enrichplot’, and ‘clusterProfiler’ software packages in R were used to perform GO. FDR <0.05 was considered statistically significant.

### Statistics

Statistics were performed using the SPSS software (version 25.0, IBM Corp., Armonk, NY) and R language software (version 3.5.1, available at http://www.R-project.org). Normal distribution and equal variance were assessed by Kolmogorov–Smirnov and Levene tests. To evaluate inter-observer reliability for VOI drawing and radiomics analysis, intraclass correlation coefficient (ICC) test was performed. The “mRMRe”, “Glmnet”, and “pROC” packages were applied to execute mRMR algorithm, LASSO logistic regression, and ROC curves, respectively. AUCs of these models were compared by Delong test. *P* < 0.05 was considered statistically significant.

## Results

### CD8A Correlated With Other Immune Expression Profiles in TCGA Cohort

**Figure 2:**
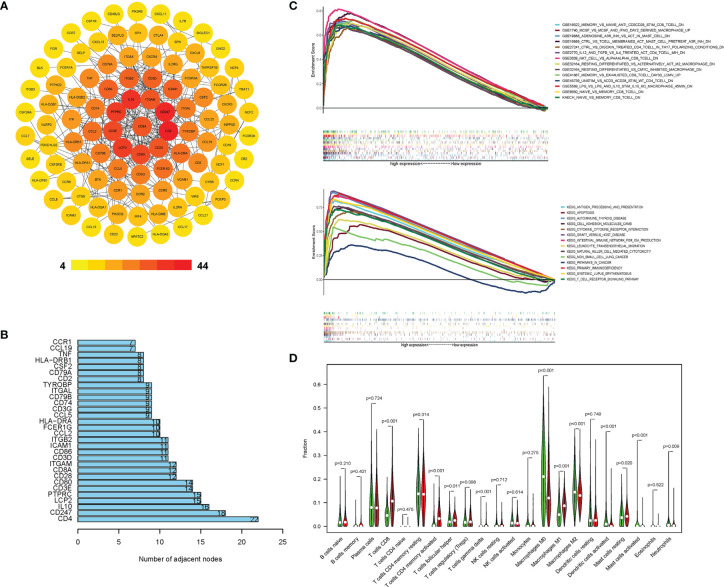
CD8A correlated with other immune expression profiles in TCGA cohort. **(A)** PPI network of DEGs with integrated scores > 0.20, in which top 30 central genes were obtained **(B)**. The difference in KEGG, immunologic signature **(C)**, and immune cells’ proportion **(D)** between the CD8-high and CD8-low groups.

The results showed that CD8A was one of the immune score-related DEGs (**Supplementary Figure S1**). A PPI network was constructed, which consisted of 200 nodes. **Figure 2A** depicts the subnetwork containing the most nodes and edges, and top 30 genes were identified in PPI network (**Figure 2B**). After PPI network was loaded into the cell landscape, CD8A was located in the top 10 nodes, and the PPI network of CD8A was composed of genes/proteins involved in immunomodulation, which indicates that CD8A plays an important role in tumor immune regulation. Further, GSEA was performed to investigate the differences between CD8-high group and CD8-low group in enrichment pathways and immunologic signatures. As **Figure 2C** shows, in CD8-high group, pathways were primarily associated with innate immune and immune response systems (**Table S1**). Immunologic signatures in CD8-high group were mainly related to regulatory genes of immune cells (**Table S2**). Furthermore, 22 immune cells were analyzed to explore the differences in immune infiltration using Cibersort between these two groups. Significant differences between CD8-high and CD8-low groups were shown in CD8 T cells, follicular helper T cells, gamma delta T cells, activated CD4 memory T cells, macrophages, resting and activated Mast cells, activated dendritic cells, and neutrophils (**Figure 2D**). These findings indicate that CD8 expression signature could represent the TIME profiles. Therefore, CD8 was chosen to be the marker to distinguish ‘hot’ and ‘cold’ TIME in this study.

### Prediction of Immune Profiles by PET/CT Radiomics and Clinical Characteristics in DPH Cohort

#### Clinical Characteristics

**Table S3** demonstrates the patients’ characteristics in DPH cohort. Significant differences were found in gender, smoking history, and pathology between CD8-high and CD8-low groups in both training and validation sets (**Table S3**). The imaging features, including tumor maximum length and SUVmax, were shown to be significantly longer/higher in CD8-high group relative to CD8-low group (both *P* < 0.001).

#### Feature Selection in the Three Radiomics Models

**Figure 3:**
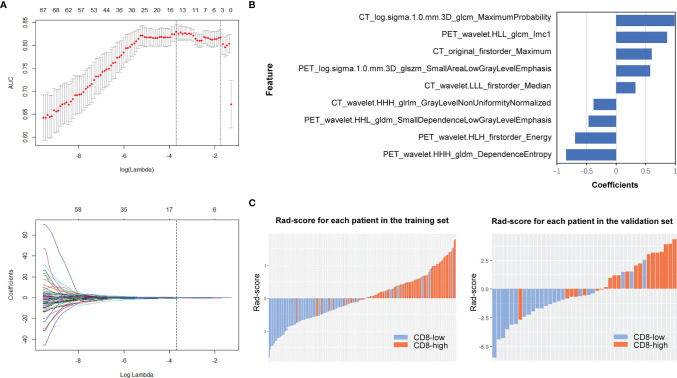
PET/CT radiomics feature selection and model construction. In PET/CT model, **(A)** LASSO method was calculated. **(B)** The final retained features after selection and their corresponding coefficients. **(C)** Rad-score of each patient in the training and validation sets.

A total of 1748 radiomic features were extracted. The ICC values ranging from 0.82 to 0.98 suggested high reliability of VOI drawing and great consistency between two readers. For PET/CT model, 70 features were retained after mRMR analysis, and nine optimal features were identified for constructing the model by LASSO logistic regression (**Figures 3A, B**; **Table S4**). Whereas, for PET or CT model, seven or four features were retained for constructing the model (**Figures S2, S3**; **Tables S5, S6**). **Figure 3C** shows the rad-score distribution of each patient in both training and validation groups, which indicated NSCLCs with CD8-high expression had higher rad-score than those with CD8-low expression.

#### Performance of the Radiomics Models and Radiomics-Clinical Combined Model

**Figure 4:**
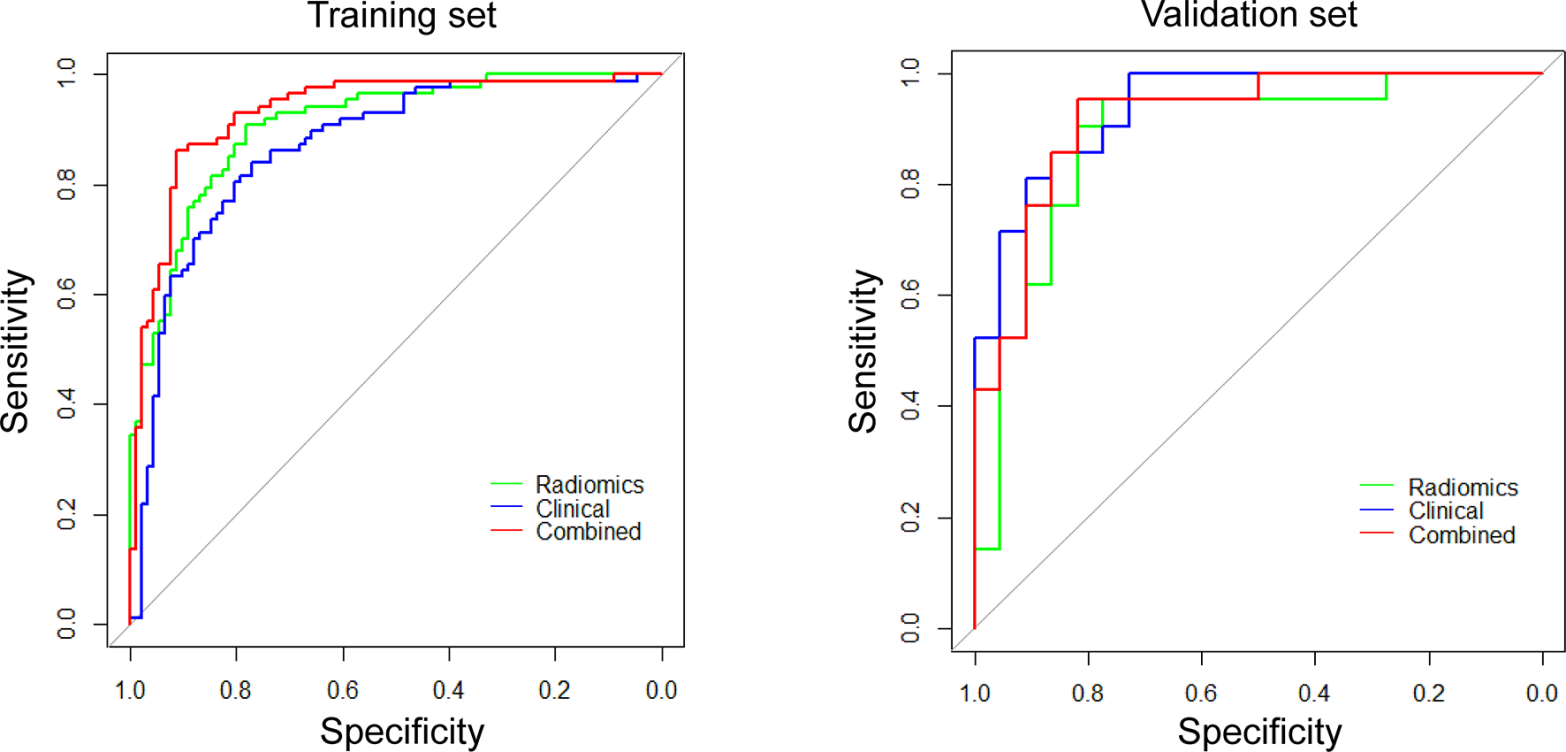
ROC curves for differentiating CD8 expression status of these three models (radiomics, clinical, and combined models).

The ROC curves and predictive performance of the three radiomics models are shown in **Figure S4** and **Table S7**. Delong test demonstrated that PET/CT model outperformed CT model to predict CD8 expression significantly (Z = 2.1518, *P* = 0.0314). After clinical model screening, gender, smoking, pathology, stage, SUVmax, maximum length, bur, calcification, and ground glass were associated with CD8 expression by univariate logistic analysis (**Table S8**). Subsequently, we further analyzed the nine clinical features using multivariate logistic and found four clinical variables (gender, pathology, SUVmax, ground glass) with significant influence on the model, among which two clinical features (gender, SUVmax) were independent predictors of CD8 expression (**Table S9**). Then, we analyzed the differences in gender between the CD8-high and CD8-low groups in NSCLCs from TCGA, which showed significant differences in gender between these two groups (**Table S10**). Further, we established PET/CT radiomics-clinical combined model to evaluate the TIME profiles by integrating the significantly associated clinical features and Rad-score. The results revealed that the combined model could perfectly predict TIME status in NSCLC (training: AUC = 0.932, testing: AUC = 0.920), which showed better performance than both clinical model (AUC = 0.868, Z = 2.9107, *P* = 0.0036) and radiomics model (AUC = 0.907, Z = 2.1363, *P* = 0.0326) (**Figure 4**; **Table S7**).

#### Construction and Validation of the Individualized Nomogram

**Figure 5:**
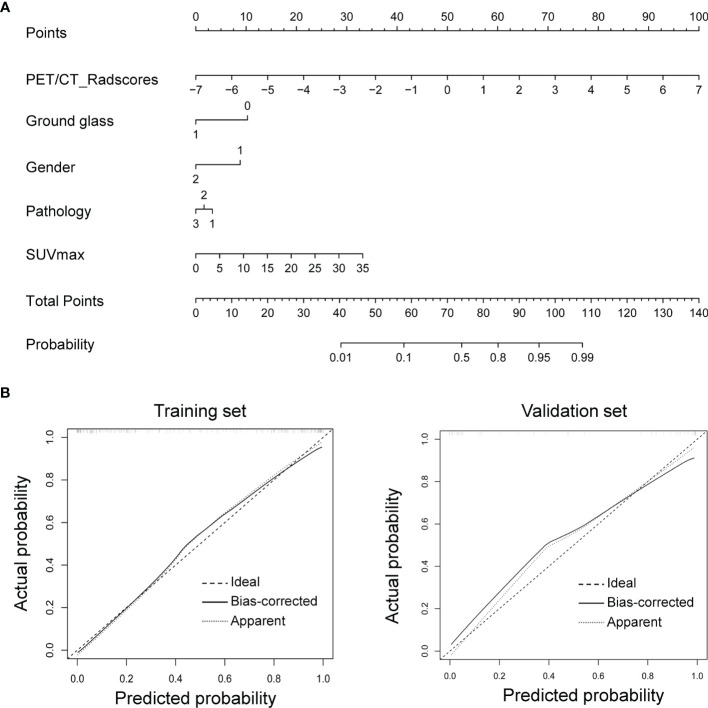
Nomogram for CD8 expression status prediction. **(A)** Nomogram integrating clinical features with Rad-score. **(B)** The nomogram’s calibration was assessed using calibration curves, which was confirmed by Hosmer–Lemeshow test in the training (left) and validation sets (right).

Given that the radiomics-clinical combined model performed better at predicting TIME status in NSCLC, we drew the nomogram integrating PET/CT Rad-score, ground glass, gender, pathology, and SUVmax for establishing a risk-scoring model (**Figure 5A**). The calibration curve showed predicted value was in accordance with the observed value in both training and validation sets (**Figure 5B**). No significant difference was observed on the Hosmer–Lemeshow test in either the training (*P* = 0.6774) or validation sets (*P* = 0.4759), which indicated a good fit.

### Application of the Radiomics-Clinical Combined Model in TCIA Cohort

**Figure 6:**
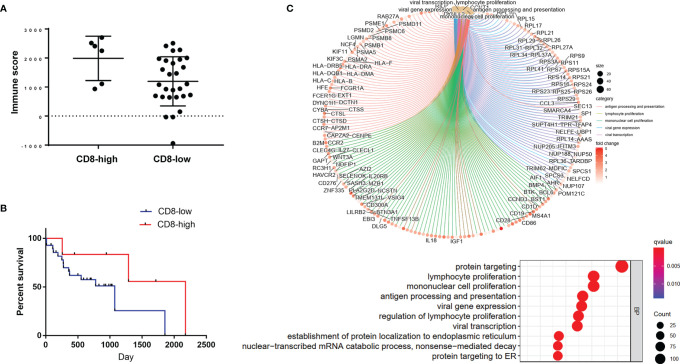
Application of the radiomics-clinical combined model in TCIA cohort. **(A)** Immune scores of the predicted CD8-high and CD8-low groups. **(B)** The overall survival curve of these two predicted groups. **(C)** The enrichment relationship between genes and the main enriched terms (up) and the top 10 BP terms (bottom) in GO analysis.

We applied the radiomics-clinical combined model in TCIA cohort to predict the TIME phenotypes. Based on the combined model, patients in TCIA cohort were classified into predicted CD8-high and predicted CD8-low groups. The predicted CD8-high group showed significantly higher immune scores and more activated immune pathways than the predicted CD8-low group (*P* = 0.0421, 95% CI: -1558 to -30.21; **Figures 6A, C**). Then, we attempted to assess whether the predicted TIME correlated with overall survival. Overall survival in both predicted groups showed no significant difference (*P* = 0.2194, HR: 0.3944, 95% CI: 0.1237 to 1.258, **Figure 6B**). GO analysis (**Figure 6C**) demonstrated that the top five enriched BP terms were viral gene expression, viral transcription, lymphocyte proliferation, mononuclear cell proliferation, and antigen processing and presentation.

## Discussion

Given the increasing use of immunotherapy in cancers, knowing the individual’s TIME phenotypes could help identify the patients who will respond to immunotherapy. Herein, we successfully developed a model based on machine learning to evaluate TIME profiles by combining ^18^F-FDG PET/CT radiomics data with clinical features, with an excellent performance. When we applied our model in TCIA cohort, the predicted CD8-high group had significantly higher immune scores and more activated immune pathways than the predicted CD8-low group. These findings implicate that the approach to estimate TIME phenotypes of individual tumor lesions by ^18^F-FDG PET/CT radiomics could be potentially feasible in clinics.

Recent studies reported three distinct TIME phenotypes, including immune-inflamed, immune-excluded, and immune-desert (6, 26). Immune-inflamed phenotype is characterized by high infiltration of CD8+ CTLs with upregulated IFN γ signal pathway, and expression of immune cell checkpoint markers (26–28). Tumors with this TIME phenotype are considered immunologically ‘hot’ tumors, which tend to respond to immunotherapy (29–31). While, low infiltration of CD8+ CTLs is the characteristic of the two other TIME phenotypes, immune-excluded and immune-desert, which are considered not to be inflamed, called immunologically ‘cold’ tumors (9, 32, 33). Thus, CD8+ CTL was chosen to be the marker to distinguish ‘hot’ and ‘cold’ TIME in this study, which was also applied in other previous reports (18, 34). Immune infiltration was estimated by CD8A gene expression and CD8 immunohistochemistry, which was consistent with the previous study that revealed the correlation of CD8A gene with the CD8 cells infiltration (35). In addition, a recent study revealed that CD8A was positively correlated with the immune score in almost all 33 tumor types (36). Furthermore, in our study, the TCGA results showed that CD8A was one of the immune score-related DEGs. After PPI network loaded into the cell landscape, CD8A was located in the top 10 nodes, and the PPI network of CD8A composed of genes/proteins involved in immunomodulation, which indicates that CD8A plays an important role in tumor immune regulation. Therefore, we chose CD8A and analyzed whether CD8A could represent the TIME profiles in this study. Consistent with these previous studies, we found CD8 expression signature could represent the TIME phenotypes in1037 NSCLC patients from TCGA.

Given the important role of TIME in immunotherapy, various methods were currently being used to measure TIME. Pepe F et al. adopted cytological samples for tumor immune-checkpoint biomarkers assessment in patients with lung cancer, and found that cytological samples yielded more accurate results than traditional histological matched specimens (37). However, the preoperative preparation phase of cytology was complicated, and was an invasive examination. Thus, emerging research focuses on using non-invasive imaging to predict the TIME phenotypes (21, 38–41). Recently, based on CT images, some machine learning models were reported to discriminate tumors with ‘hot’ versus ‘cold’ TIME, and were also used for prediction of immunotherapy efficacy in cancers (18, 42). In addition, the metabolic pattern of the tumor microenvironment was estimated by ^18^F-FDG PET/CT (21–24). Research has revealed a relationship between metabolic status and immune cells in TME. If machine learning is used to reflect the heterogeneity of TIME in PET/CT images, it may have greater clinical value. Therefore, we developed a ^18^F-FDG PET/CT radiomics model to evaluate TIME profiles.

In this study, the predictive power of PET/CT radiomics model was better than that of CT radiomics model to define TIME phenotypes in NSCLC. Further, PET/CT radiomics-clinical combined model, which consisted of gender, pathology, ground glass composition, SUVmax, and Rad-score was established. The combined model outperformed the radiomics model or clinical model alone. In our study, significant differences were found in gender between CD8-high and CD8-low groups, which was consistent with the previous study (43) and results of TCGA cohort. In clinical features, SUVmax had been previously reported positively correlated with CD8+ CTLs in tumors, which was consistent with our study (21, 44, 45). In addition, tumors with ground glass composition were found more probable to be immunologically ‘cold’ tumors. In PET/CT radiomics, three of selected nine radiomics features are first-order radiomics features. According to Lin et al., the first-order radiomics features were reported robust and repeatable, thus they could accurately provide intensity-based indexes of lesions (46). The remaining six radiomics features are all related to image heterogeneity and uniformity. The results indicated that NSCLCs with ‘hot’ TIME were more heterogeneous, compared to ‘cold’ TIME. Taken together, PET/CT radiomics-clinical combined model could predict TIME status in NSCLCs with high performance.

Our study has some limitations. First, although the predictive performance of this combined model was applied in independent cohort from TCIA, the sample size is small. Thus, it is necessary to validate this model in large-scale prospective studies and, in the future, apply in independent multi-cohortsSecond, we discriminated the TIME phenotypes based on CD8 expression signature, which were also used in other studies (18, 34). Prospective studies should include additional TIME subtypes to understand the tumor immune functions (47, 48).

## Conclusion

In summary, this study highlights that ^18^F-FDG PET/CT radiomics-clinical combined model could be a clinically practical approach to non-invasively detect TIME phenotypes in NSCLCs. In spite of the necessity to validate this model in large-scale prospective studies, these findings indicate the potential for non-invasive biomarker development in immunotherapy.

## Data Availability Statement

The datasets presented in this study can be found in online repositories. The names of the repository/repositories and accession number(s) can be found in the article/**Supplementary Material**.

## Ethics Statement

The studies involving human participants were reviewed and approved by the Ethical Committee of Daping Hospital, Army Medical University. Written informed consent for participation was not required for this study in accordance with the national legislation and the institutional requirements.

## Author Contributions

XC and KL conceived and designed the study. JS, RX, and WZ collected the data of PET/CT and clinical data. HT and XC analyzed TCGA and TCIA data. JS and MZ extracted PET/CT radiomics features. HL analyzed PET/CT radiomics data. JF and RX analyzed PET/CT imaging feature. KL, HT, and HL performed statistics. XC and JF performed figures and tables preparation. XC wrote the manuscript. JF and KL revised the manuscript. All authors revised the manuscript, read and approved the submitted version.

## Funding

This work was supported by grants from Science and Technology Innovation Ability Enhancement Project of Army Medical University (2019XLC3054), the Natural Science Foundation of Chongqing (cstc2019jcyj-msxmX0123), Talent Innovation Ability Training Program of Daping Hospital (2019CXLCC010), the National Natural Science Foundation of China (81801672), and Chongqing Clinical Research Centre of Imaging and Nuclear Medicine (CSTC2015YFPT-gcjsyjzx0175).

## Conflict of Interest

Author HL was employed by GE Healthcare.

The remaining authors declare that the research was conducted in the absence of any commercial or financial relationships that could be construed as a potential conflict of interest.

## Publisher’s Note

All claims expressed in this article are solely those of the authors and do not necessarily represent those of their affiliated organizations, or those of the publisher, the editors and the reviewers. Any product that may be evaluated in this article, or claim that may be made by its manufacturer, is not guaranteed or endorsed by the publisher.
