# Supplementary material for: A Machine Learning Model Based on PET/CT Radiomics and Clinical Characteristics Predicts Tumor Immune Profiles in Non-Small Cell Lung Cancer: A Retrospective Multicohort Study
Source: Front Immunol. 2022 Apr 29;13:859323. doi: 10.3389/fimmu.2022.859323 (PMC9105942; doi:10.3389/fimmu.2022.859323)
Supplement: Supplementary file 1 [file DataSheet_1.docx]

Supplementary Material

**Supplementary Materials and methods**

**Immunohistochemistry**

For each case of the immune phenotype dataset, all paraffin blocks obtained from surgery were used for immunohistochemistry. Tissue slide (4 μm) were obtained from each block, followed by standard immunohistochemistry, using primary antibody anti-CD8 (Jinqiao Bio) overnight at 4 ℃. The secondary reactions were visualized with diaminobenzidine. Two independent pathologists blinded to clinical data selected five non-overlapping and non-contiguous areas for each slide. Assessment of CD8+ TIL density was performed according to established protocols (1). In assessing the density of CD8+ TILs, the cut-off for stratifying subgroups was the median value (2).

**Supplementary Results**

**Identification of TIME DEGs**

The RNA-Seq data and clinical information for 1137 patients with NSCLC were downloaded from TCGA and tumor samples were evaluated using the ESTIMATE algorithm. Immune scores ranged from -942.774 to 2706.266. According to the median scores, all 1137 samples were divided into high- and low-score groups, and the relationship between immune score and clinical characteristics were analyzed. The OS of patients with NSCLC showed no significant correlation with immune score (*P* = 0.212). By comparing the gene expression levels of the high- and low-scoring groups, we screened DEGs with |log2FC| > 1.0 and FDR < 0.05, and displayed them as a heatmap (Figure S4). Statistical analysis identified 901 significantly upregulated and 440 significantly downregulated DEGs in the immune score group.

**Reference**

1. Salgado R, Denkert C, Demaria S, Sirtaine N, Klauschen F, Pruneri G, et al. The evaluation of tumor-infiltrating lymphocytes (TILs) in breast cancer: recommendations by an International TILs Working Group 2014. *Ann Oncol* (2015) 26(2):259-71. doi:10.1093/annonc/mdu450.

2. Sun R, Limkin EJ, Vakalopoulou M, Dercle L, Champiat S, Han SR, et al. A radiomics approach to assess tumour-infiltrating CD8 cells and response to anti-PD-1 or anti-PD-L1 immunotherapy: an imaging biomarker, retrospective multicohort study. *Lancet Oncol* (2018) 19(9):1180-91. doi:10.1016/S1470-2045(18)30413-3.

Table S1. The difference in enrichment pathways between CD8-high and CD8-low groups.

| NAME | FDR q-value |
| --- | --- |
| KEGG_NATURAL_KILLER_CELL_MEDIATED_CYTOTOXICITY | <0.001 |
| KEGG_CHEMOKINE_SIGNALING_PATHWAY | <0.001 |
| KEGG_T_CELL_RECEPTOR_SIGNALING_PATHWAY | <0.001 |
| KEGG_CYTOKINE_CYTOKINE_RECEPTOR_INTERACTION | <0.001 |
| KEGG_CELL_ADHESION_MOLECULES_CAMS | <0.001 |
| KEGG_TOLL_LIKE_RECEPTOR_SIGNALING_PATHWAY | <0.001 |
| KEGG_VIRAL_MYOCARDITIS | <0.001 |
| KEGG_B_CELL_RECEPTOR_SIGNALING_PATHWAY | <0.001 |
| KEGG_ANTIGEN_PROCESSING_AND_PRESENTATION | <0.001 |
| KEGG_NATURAL_KILLER_CELL_MEDIATED_CYTOTOXICITY | <0.001 |

| NAME | FDR q-value |
| --- | --- |
| GSE5589_LPS_VS_LPS_AND_IL10_STIM_IL10_KO_MACROPHAGE_45MIN_DN | <0.001 |
| GSE3039_NKT_CELL_VS_ALPHAALPHA_CD8_TCELL_DN | <0.001 |
| GSE16522_MEMORY_VS_NAIVE_ANTI_CD3CD28_STIM_CD8_TCELL_DN | <0.001 |
| GSE2770_IL12_AND_TGFB_VS_IL4_TREATED_ACT_CD4_TCELL_48H_DN | <0.001 |
| GSE45739_UNSTIM_VS_ACD3_ACD28_STIM_WT_CD4_TCELL_DN | <0.001 |
| GSE19888_CTRL_VS_TCELL_MEMBRANES_ACT_MAST_CELL_PRETREAT_A3R_INH_DN | <0.001 |
| GSE41867_MEMORY_VS_EXHAUSTED_CD8_TCELL_DAY30_LCMV_UP | <0.001 |
| GSE1740_MCSF_VS_MCSF_AND_IFNG_DAY2_DERIVED_MACROPHAGE_UP | <0.001 |
| GSE27241_CTRL_VS_DIGOXIN_TREATED_CD4_TCELL_IN_TH17_POLARIZING_CONDITIONS_DN | <0.001 |
| GSE19888_ADENOSINE_A3R_INH_VS_ACT_IN_MAST_CELL_DN | <0.001 |

Table S2. The difference in immunologic signatures between CD8-high and CD8-low groups.

| Clinical features | Training Cohort (n=178) | | |  | Validation Cohort (n=43) | | |
| --- | --- | --- | --- | --- | --- | --- | --- |
|  | CD8-low | CD8-high | P value |  | CD8-low | CD8-high | P value |
| Age | 62.00(55.20, 69.00) | 65.00(56.20, 69.00) | 0.387 |  | 63.00(55.00, 68.25) | 65.00(60.00, 71.50) | 0.423 |
| Gender |  |  |  |  |  |  |  |
| Male | 45(49.45%) | 64(73.56%) | 0.001 |  | 8(36.36%) | 20(95.24%) | <0.001 |
| Female | 46(50.55%) | 23(26.44%) |  |  | 14(63.64%) | 1(4.76%) |  |
| Smoking |  |  |  |  |  |  |  |
| Never | 53(58.24%) | 35(40.23%) | 0.016 |  | 16(72.73%) | 4(19.05%) | <0.001 |
| Current or ever | 38(41.76%) | 52(59.77%) |  |  | 6(27.27%) | 17(80.95%) |  |
| Pathology |  |  |  |  |  |  |  |
| Adenocarcinoma | 84(92.31%) | 57(65.52%) | <0.001 |  | 21(95.45%) | 10(47.62%) | 0.001 |
| SCC | 4(4.40%) | 27(31.03%) |  |  | 1(4.55%) | 9(42.86%) |  |
| Others | 3(3.30%) | 3(3.45%) |  |  | 0(0.00%) | 2(9.52%) |  |
| Stages |  |  |  |  |  |  |  |
| Ⅰ | 71(78.02%) | 28(32.18%) | <0.001 |  | 17(77.27%) | 11(52.38%) | 0.179 |
| Ⅱ | 4(4.40%) | 28(32.18%) |  |  | 2(9.09%) | 6(28.57%) |  |
| Ⅲ | 12(13.19%) | 30(34.48%) |  |  | 3(13.64%) | 3(14.29%) |  |
| Ⅳ | 4(4.40%) | 1(1.15%) |  |  | 0(0.00%) | 1(4.76%) |  |
| SUVmax | 3.51(1.71, 5.53) | 7.55(5.76, 9.59) | <0.001 |  | 3.31(1.46, 4.59) | 8.06(5.44, 10.38) | <0.001 |
| Maximum length | 2.20(1.72, 2.70) | 3.90(3.12, 5.50) | <0.001 |  | 2.10(1.69, 2.52) | 3.80(3.37, 4.73) | <0.001 |
| Leaflet |  |  |  |  |  |  |  |
| (-) | 1(1.10%) | 1(1.15%) | 1.0 |  | 0 | 0 | 1.0 |
| (+) | 90(98.90%) | 86(98.85%) |  |  | 22(100.00%) | 21(100.00%) |  |
| Bur |  |  |  |  |  |  |  |
| (-) | 15(16.48%) | 22(25.29%) | 0.148 |  | 3(13.64%) | 8(38.10%) | 0.066 |
| (+) | 76(83.52%) | 65(74.71%) |  |  | 19(86.36%) | 13(61.90%) |  |
| Pleural adhesion |  |  |  |  |  |  |  |
| (-) | 19(20.88%) | 15(17.24%) | 0.537 |  | 4(18.18%) | 4(19.05%) | 0.75 |
| (+) | 72(79.12%) | 72(82.76%) |  |  | 18(81.82%) | 17(80.95%) |  |
| Air bronchogram |  |  |  |  |  |  |  |
| (-) | 44(48.35%) | 48(55.17%) | 0.363 |  | 13(59.09%) | 10(47.62%) | 0.451 |
| (+) | 47(51.65%) | 39(44.83%) |  |  | 9(40.91%) | 11(52.38%) |  |
| Vacuole sign |  |  |  |  |  |  |  |
| (-) | 86(94.51%) | 77(88.51%) | 0.15 |  | 22(100.00%) | 21(100.00%) | 1.0 |
| (+) | 5(5.49%) | 10(11.49%) |  |  | 0 | 0 |  |
| Calcification |  |  |  |  |  |  |  |
| (-) | 89(97.80%) | 78(89.66%) | 0.024 |  | 22(100.00%) | 21(100.00%) | 1.0 |
| (+) | 2(2.20%) | 9(10.34%) |  |  | 0 | 0 |  |
| Ground glass |  |  |  |  |  |  |  |
| (-) | 78(85.71%) | 86(98.85%) | 0.001 |  | 20(90.91%) | 21(100.00%) | 0.488 |
| (+) | 13(14.29%) | 1(1.15%) |  |  | 2(9.09%) | 0(0.00%) |  |

Table S3. Clinical characteristics of NSCLC patients in DPH dataset.

Medians (interquartile range) on behalf of age, SUVmax, and maximum length, because they did not comply with normal distribution. *p* < 0.05 showed significant difference.

Table S4. The selected most predictive subset of feature and the corresponding coefficients in PET/CT model.

| Features | Coefficient |
| --- | --- |
| PET_wavelet.HHL_gldm_SmallDependenceLowGrayLevelEmphasis | -0.46524 |
| CT_wavelet.LLL_firstorder_Median | 0.325499 |
| PET_log.sigma.1.0.mm.3D_glszm_SmallAreaLowGrayLevelEmphasis | 0.573298 |
| PET_wavelet.HLL_glcm_Imc1 | 0.858512 |
| PET_wavelet.HHH_gldm_DependenceEntropy | -0.84305 |
| CT_original_firstorder_Maximum | 0.600745 |
| CT_log.sigma.1.0.mm.3D_glcm_MaximumProbability | 0.98822 |
| CT_wavelet.HHH_glrlm_GrayLevelNonUniformityNormalized | -0.38064 |
| PET_wavelet.HLH_firstorder_Energy | -0.69209 |

Table S5. The selected most predictive subset of feature and the corresponding coefficients in PET model.

| Features | Coefficient |
| --- | --- |
| PET_wavelet.HHL_gldm_SmallDependenceLowGrayLevelEmphasis | -0.6197 |
| PET_log.sigma.1.0.mm.3D_glszm_SmallAreaLowGrayLevelEmphasis | 0.37414 |
| PET_wavelet.HHH_gldm_DependenceVariance | -0.88551 |
| PET_wavelet.HLH_firstorder_TotalEnergy | -0.62001 |
| PET_original_firstorder_10Percentile | 0.882625 |
| PET_wavelet.HHH_glszm_GrayLevelNonUniformityNormalized | -0.47422 |
| PET_wavelet.HHH_gldm_DependenceNonUniformityNormalized | 0.683836 |

Table S6. The selected most predictive subset of feature and the corresponding coefficients in CT model.

| Features | Coefficient |
| --- | --- |
| CT_wavelet.LLL_firstorder_Median | 0.36672 |
| CT_wavelet.HHH_glrlm_GrayLevelNonUniformityNormalized | -0.38661 |
| CT_log.sigma.1.0.mm.3D_glcm_MaximumProbability | 1.261705 |
| CT_wavelet.HLL_firstorder_Maximum | 0.613498 |

Table S7. The performance of five models in training and testing groups.

| Model | Training cohort (n=178) | | |  | | Test cohort (n=43) | | |
| --- | --- | --- | --- | --- | --- | --- | --- | --- |
|  | AUC (95% CI) | Sensitivity | Specificity | |  | AUC (95% CI) | Sensitivity | Specificity |
| PET model | 0.861(0.806-0.917) | 0.816 | 0.802 | |  | 0.868(0.761-0.975) | 0.905 | 0.727 |
| CT model | 0.888 (0.839-0.937) | 0.862 | 0.813 | |  | 0.868 (0.758-0.978) | 0.952 | 0.727 |
| PET/CT model | 0.907(0.865-0.950) | 0.908 | 0.780 | |  | 0.883(0.774-0.992) | 0.952 | 0.773 |
| Clinical model | 0.868(0.813-0.922) | 0.839 | 0.769 | |  | 0.939 (0.875-1.00) | 1.000 | 0.727 |
| Combined model | 0.932(0.893-0.970) | 0.862 | 0.912 | |  | 0.920(0.839-1.00) | 0.952 | 0.818 |

AUC, area under the curve; 95% CI, 95% confidence interval.

Table S8. Univariate logistic analyze of the extracted clinical features for TIME status.

| Variable | OR (95% CI) | *P*-value |
| --- | --- | --- |
| Age | 1.017 (0.986, 1.048) | 0.282 |
| Gender | 0.252 (0.141, 0.453) | <0.001 |
| Smoking | 2.774 (1.609, 4.785) | <0.001 |
| Pathology | 4.600 (2.303, 9.190) | <0.001 |
| Stage | 2.096 (1.511, 2.909) | <0.001 |
| SUVmax | 1.526 (1.348, 1.726) | <0.001 |
| Maximum length | 4.614 (3.044, 6.996) | <0.001 |
| Leaflet | 0.955 (0.059, 15.469) | 0.974 |
| Bur | 0.493 (0.255, 0.950) | 0.035 |
| Pleural adhesion | 1.197 (0.610, 2.350) | 0.601 |
| Air bronchogram | 0.877 (0.517, 1.488) | 0.628 |
| Vacuole sign | 2.204 (0.728, 6.673) | 0.162 |
| Calcification | 5.045 (1.065, 23.907) | 0.041 |
| Ground glass | 0.061 (0.008, 0.471) | 0.007 |

*P* < 0.05 showed significant difference.

Table S9. Multivariate logistic analyze of the extracted clinical features for TIME status.

| Variable | OR (95% CI) | *P*-value |
| --- | --- | --- |
| Gender | 0.453 (0.227, 0.904) | 0.025 |
| Pathology | 1.726 (0.831, 3.587) | 0.143 |
| SUVmax | 1.424 (1.249, 1.624) | <0.001 |
| Ground glass | 0.003 (0.00,4929.931) | 0.423 |

*P* < 0.05 showed significant difference.

Table S10. The difference in gender between the CD8 high and low-expression groups in TCGA cohort

| Group | Female | Male | *P*-value |
| --- | --- | --- | --- |
| CD8-low | 174 | 318 | 0.0018 |
| CD8-high | 222 | 270 |  |

*P* < 0.05 showed significant difference.

**Supplementary Figure Legends**

**Figure S1** The heatmap shows the DEGs between the high immune score group and the low immune score group. The color indicates the fold change of gene expression, the greater the change is, the darker the color (red is up, blue is down). CD8A is the upregulated gene related to immune score.

**Figure S2** PET radiomics feature selection and model construction. (A) Calculation of the LASSO logistic regression algorithm in PET model. (B) The selected most predictive subset of feature and the corresponding coefficients in PET model. (C) Rad-score of patients in the training and validation sets.

**Figure S3** CT radiomics feature selection and model construction. (A) Calculation of the LASSO logistic regression algorithm in CT model. (B) The selected most predictive subset of feature and the corresponding coefficients in CT model. (C) Rad-score of patients in the training and validation sets.

**Figure S4** ROC curves for the PET, CT, and PET/CT radiomics models in differentiating CD8 expression status.
