# Supplementary figures and images for: A Machine Learning Model Based on PET/CT Radiomics and Clinical Characteristics Predicts Tumor Immune Profiles in Non-Small Cell Lung Cancer: A Retrospective Multicohort Study
Source: Front Immunol. 2022 Apr 29;13:859323. doi: 10.3389/fimmu.2022.859323 (PMC9105942; doi:10.3389/fimmu.2022.859323)

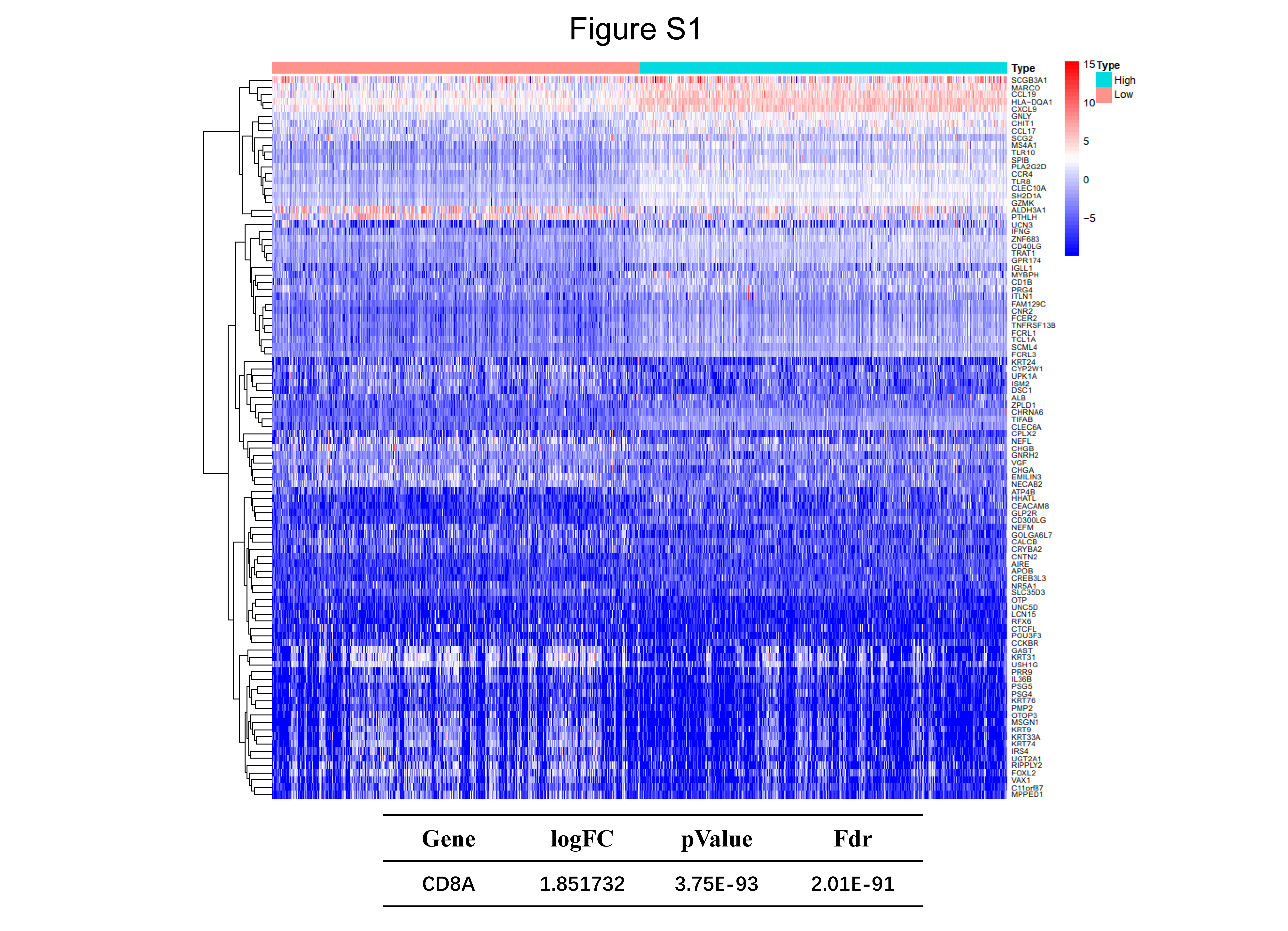

Supplement: Supplementary file 2 [file Image_1.tiff]

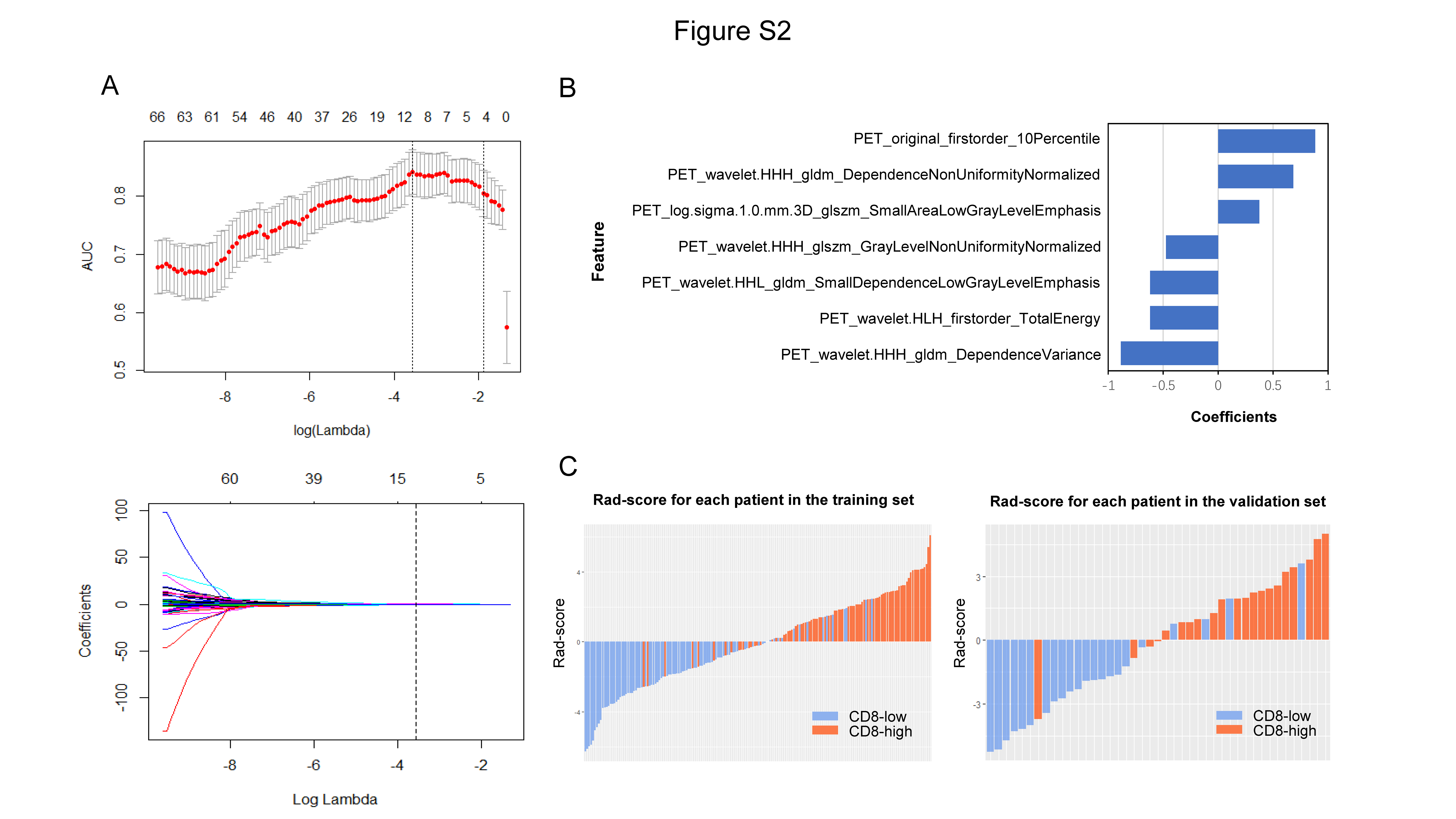

Supplement: Supplementary file 3 [file Image_2.tiff]

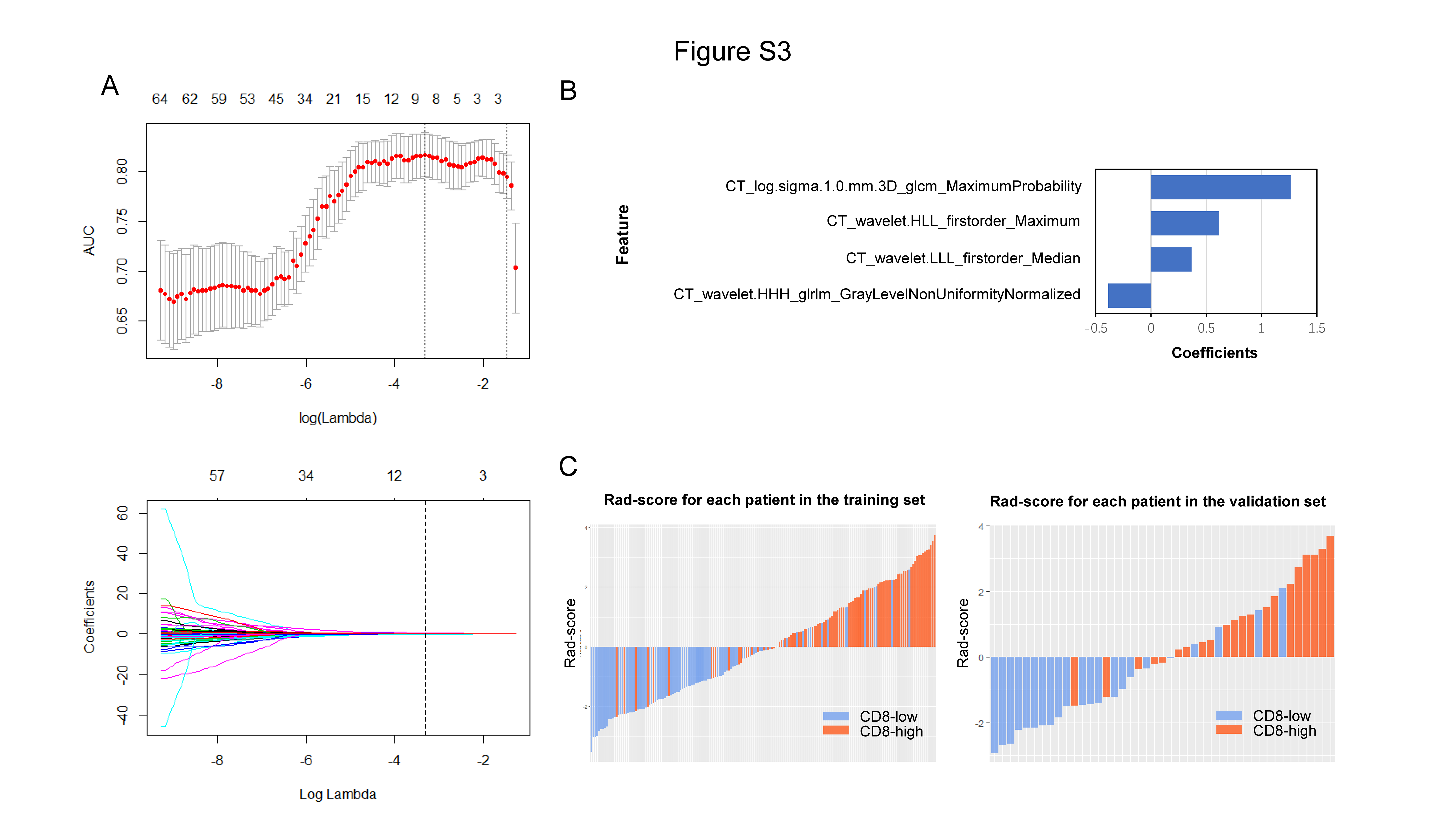

Supplement: Supplementary file 4 [file Image_3.tiff]

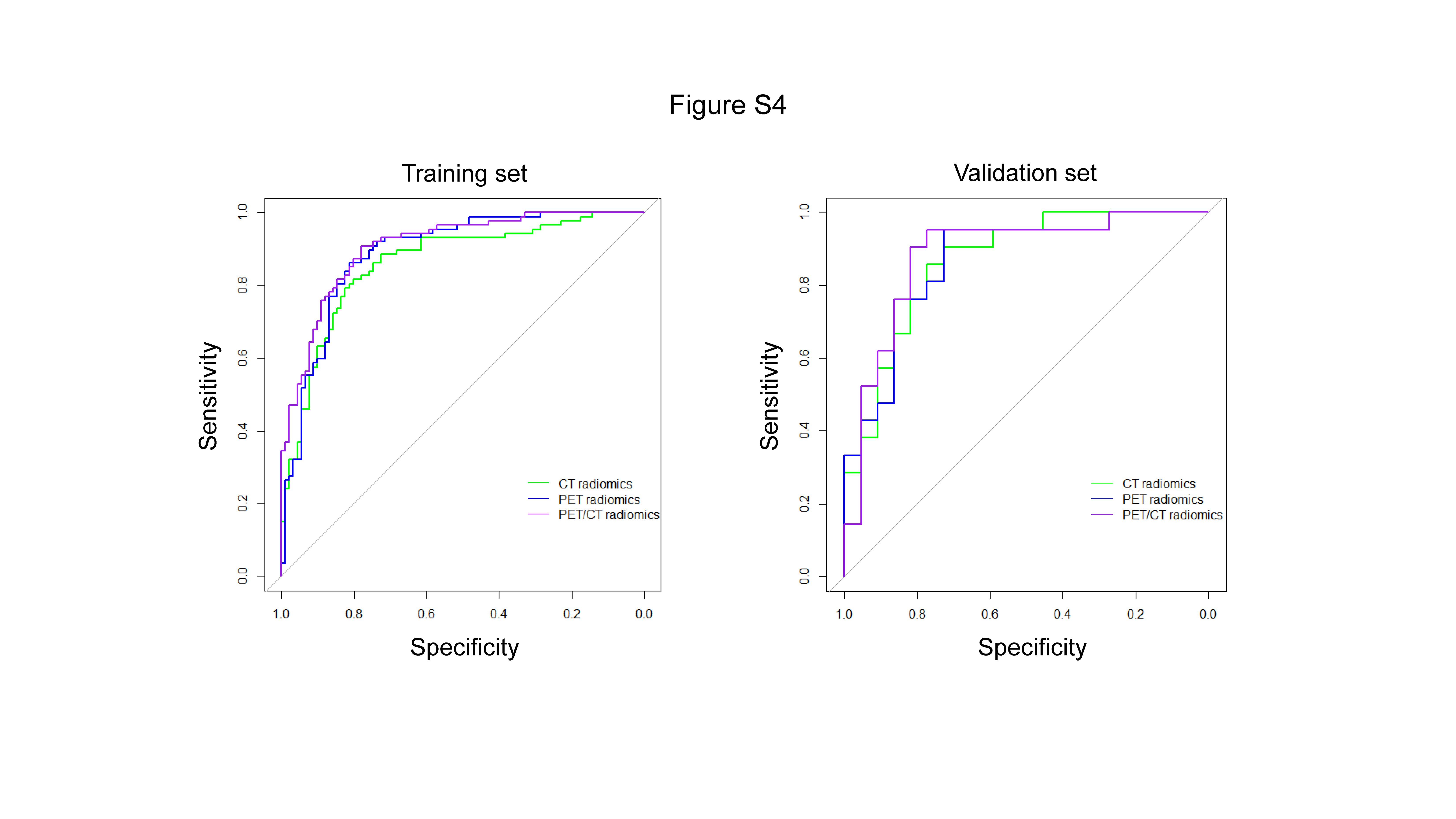

Supplement: Supplementary file 5 [file Image_4.tiff]
